# Supplementary material for: Management of Patients with Newly Diagnosed Desmoid Tumors in a First-Line Setting
Source: Cancers (Basel). 2022 Aug 12;14(16):3907. doi: 10.3390/cancers14163907 (PMC9405618; doi:10.3390/cancers14163907)
Supplement: Supplementary file 1 [file cancers-14-03907-s001.zip › cancers-1849282-supplementary.pdf]

Management of Patients with Newly Diagnosed Desmoid Tumors in the First-line Setting

Stefano Testa[1*], Nam Q. Bui[2], Gregory W. Charville[3], Raffi S. Avedian[4], Robert Steffner[4], Pejman Ghanouni[5], David G. Mohler[4], Kristen N. Ganjoo[2*]

[1] Department of Medicine, Stanford University, Stanford, CA 94304, USA, testa21@stanford.edu
[2] Division of Oncology, Department of Medicine, Stanford University, Stanford, CA 94304, USA, nambui@stanford.edu, kganjoo@stanford.edu
[3] Department of Pathology, Stanford University, Stanford, CA 94304, USA, gwc@stanford.edu
[4] Department of Orthopedic Surgery, Stanford University, Redwood City, CA 94063, USA, ravedian@stanford.edu, rsteffner@stanford.edu, dmohler@stanford.edu
[5] Department of Radiology, Stanford University, Stanford, CA 94304, USA, ghanouni@stanford.edu

* Correspondence: testa21@stanford.edu (S.T.); kganjoo@stanford.edu (K.N.G)

Table of Contents

		Page
Figure S1	Frequency distribution of specific first-line treatments	3
Figure S2	Pattern of first-line treatment choice over time	4
Figure S3	Progression-free survival (PFS) among patients with desmoid tumors treated with surgery alone, surgery plus adjuvant radiotherapy or surgery plus adjuvant systemic therapy	5
Figure S4	Stratified progression-free survival analysis: methotrexate-vinblastine versus other chemotherapy; leuprolide with or without NSAIDs versus tamoxifen with or without NSAIDs; imatinib with or without radiotherapy versus sorafenib; Cryoablation versus MR-HIFU	6
Table S1	Progression-free survival rates after first-line treatment for treatments in Figure S4	7
Figure S5	Time to next treatment (TTNT) among patients with desmoid tumors treated with surgery alone, surgery plus adjuvant radiotherapy or surgery plus adjuvant systemic therapy	8
Figure S6	Stratified time to next treatment (TTNT) analysis: methotrexate-vinblastine versus other chemotherapy; leuprolide with or without NSAIDs versus tamoxifen with or without NSAIDs; imatinib with or without radiotherapy versus sorafenib; Cryoablation versus MR-HIFU	9
Table S2	Time to next treatment after first-line treatment for treatments in Figure S6	10
Figure S7	Distribution of tumor site in each treatment group	11
Figure S8	First-line treatment based on tumor location	12
Table S3	Progression-free survival after first-line treatment in patients with desmoid tumors of the extremities	13
Table S4	Time to next treatment after first-line therapy in patients with desmoid tumors of the extremities	14
Table S5	Progression-free survival after first-line treatment in patients with desmoid tumors of the abdominal wall	15
Table S6	Time to next treatment after first-line therapy in patients with desmoid tumors of the abdominal wall	16
Table S7	Progression-free survival after first-line treatment in patients with intra-abdominal desmoid tumors	17
Table S8	Time to next treatment after first-line therapy in patients with intra-abdominal desmoid tumors	18
Figure S9	Progression-free survival (PFS) and time to next treatment (TTNT) after first line treatment in desmoid tumors of the chest wall and head and neck	19
Table S9	Progression-free survival after first-line therapy in patients with desmoid tumors of the chest wall	20
Table S10	Time to next treatment after first-line therapy in patients with desmoid tumors of the chest wall	21
Table S11	Progression-free survival after first-line therapy in patients with desmoid tumors of the head and neck	22
Table S12	Time to next treatment after first-line therapy in patients with desmoid tumors of the head and neck	23

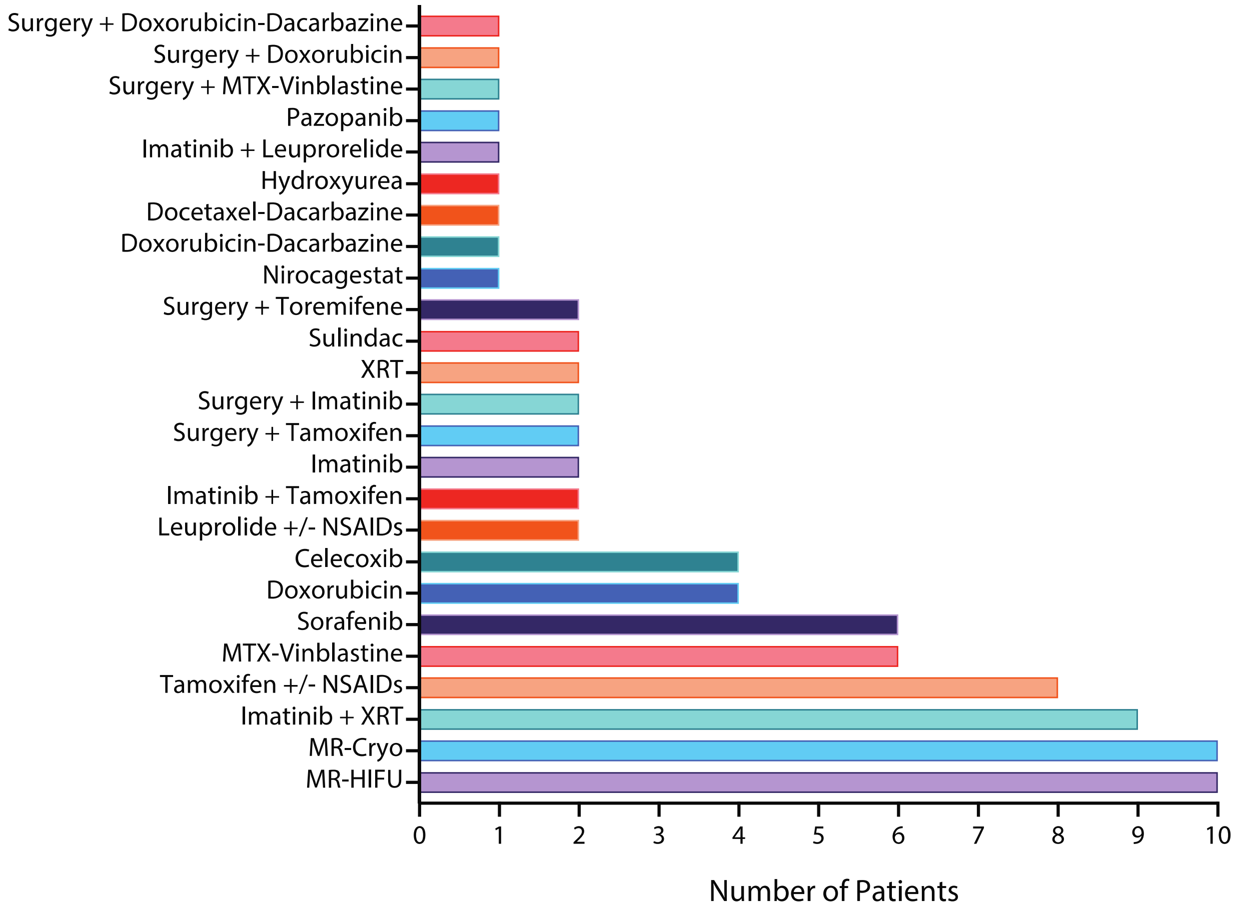

Figure S1. Frequency distribution of specific first-line treatments. Patients that received surgery alone or surgery plus adjuvant radiotherapy or that were managed expectantly are not included.

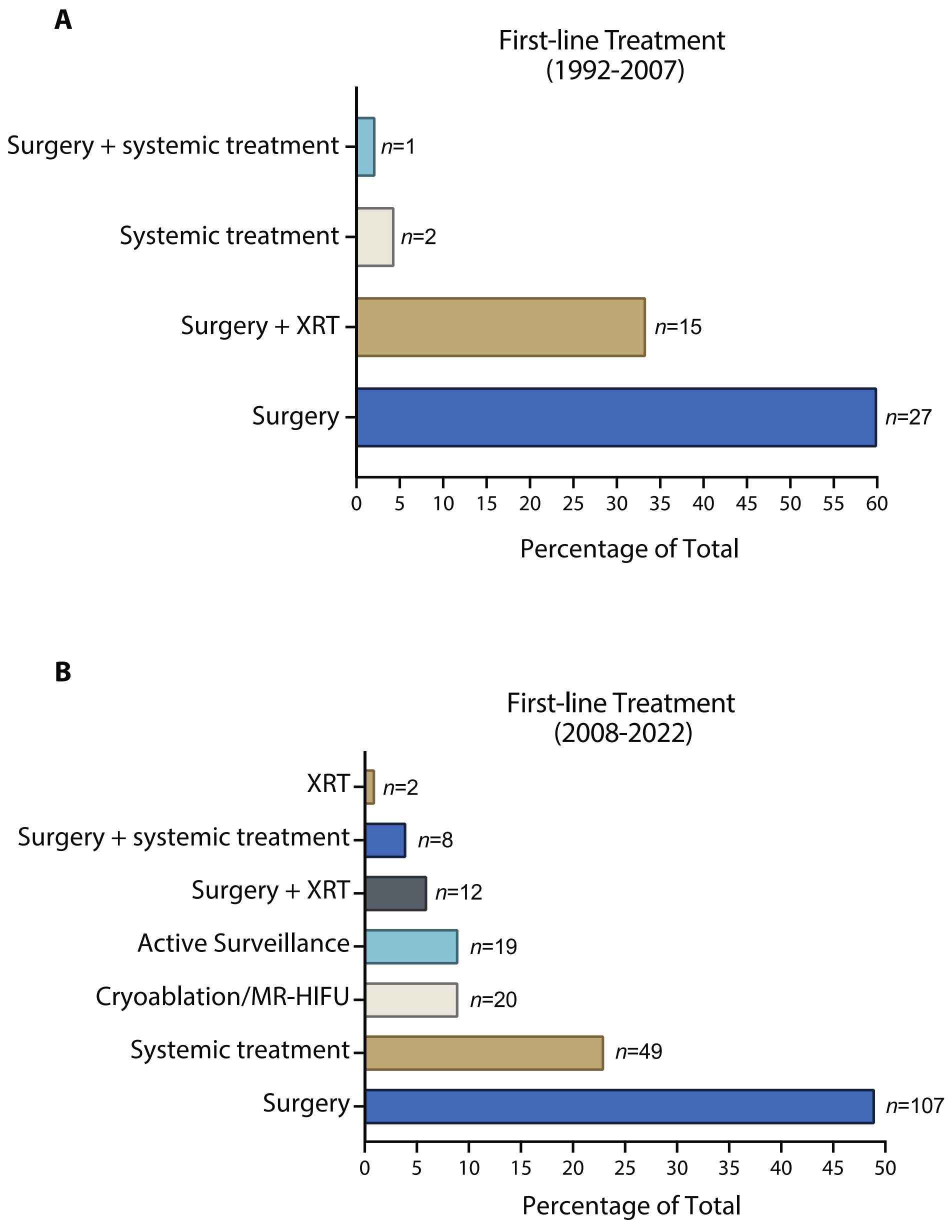

Figure S2. Pattern of first-line treatment choice over time. (A) Bar graph shows percentage of patients diagnosed between 1992 and 2007 that were treated with first-line surgery, surgery plus adjuvant radiotherapy (XRT), systemic treatment or surgery plus adjuvant systemic therapy. (B) Bar graph shows percentage of patients diagnosed between 1992 and 2007 that were treated with first-line surgery, surgery plus adjuvant radiotherapy (XRT), surgery plus adjuvant systemic therapy, radiotherapy alone, cryoablation or MR-HIFU or managed with initial active surveillance. MR-HIFU: Magnetic Resonance High Intensity Focused Ultrasound.

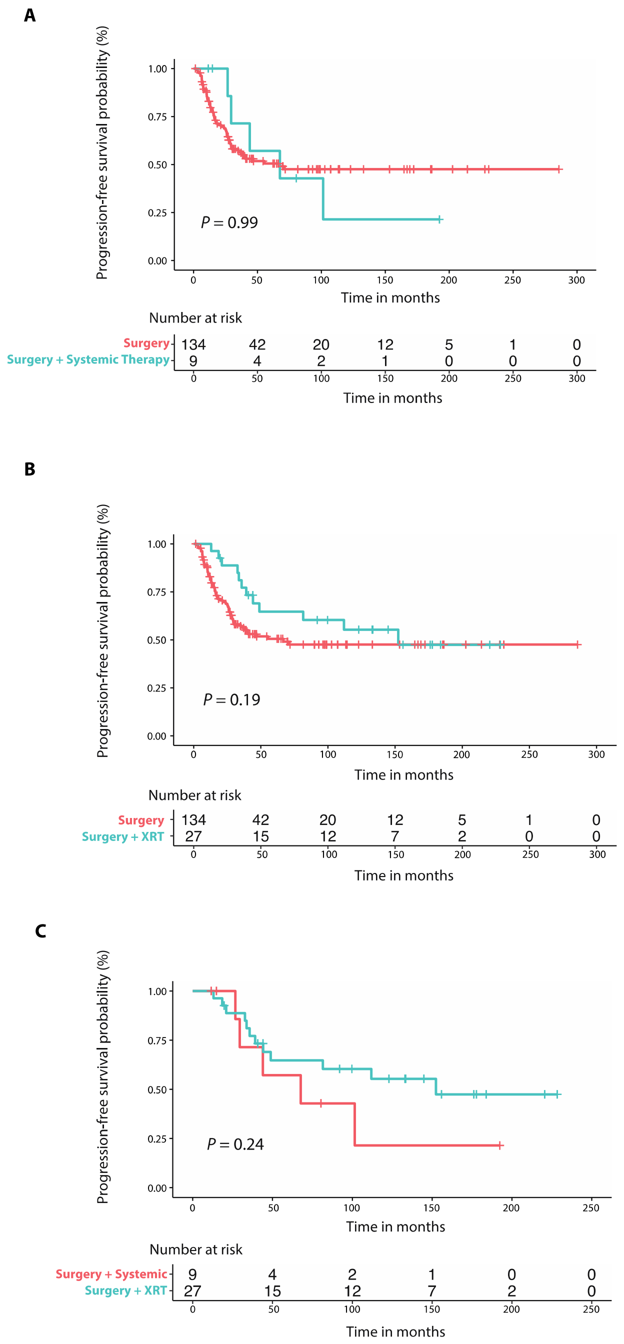

Figure S3. Progression-free survival (PFS) among patients with desmoid tumors treated with surgery alone, surgery plus adjuvant radiotherapy or surgery plus adjuvant systemic therapy. (A) Kaplan-Meier curves show PFS of patients treated with surgery compared to patients treated with surgery plus adjuvant systemic therapy. (B) Kaplan-Meier curves show PFS of patients treated with surgery compared to patients treated with surgery plus adjuvant radiotherapy. (C) Kaplan-Meier curves show PFS of patients treated with surgery plus adjuvant systemic therapy compared to surgery plus adjuvant radiotherapy. P-values were calculated with the Log-Rank method. XRT: radiotherapy.

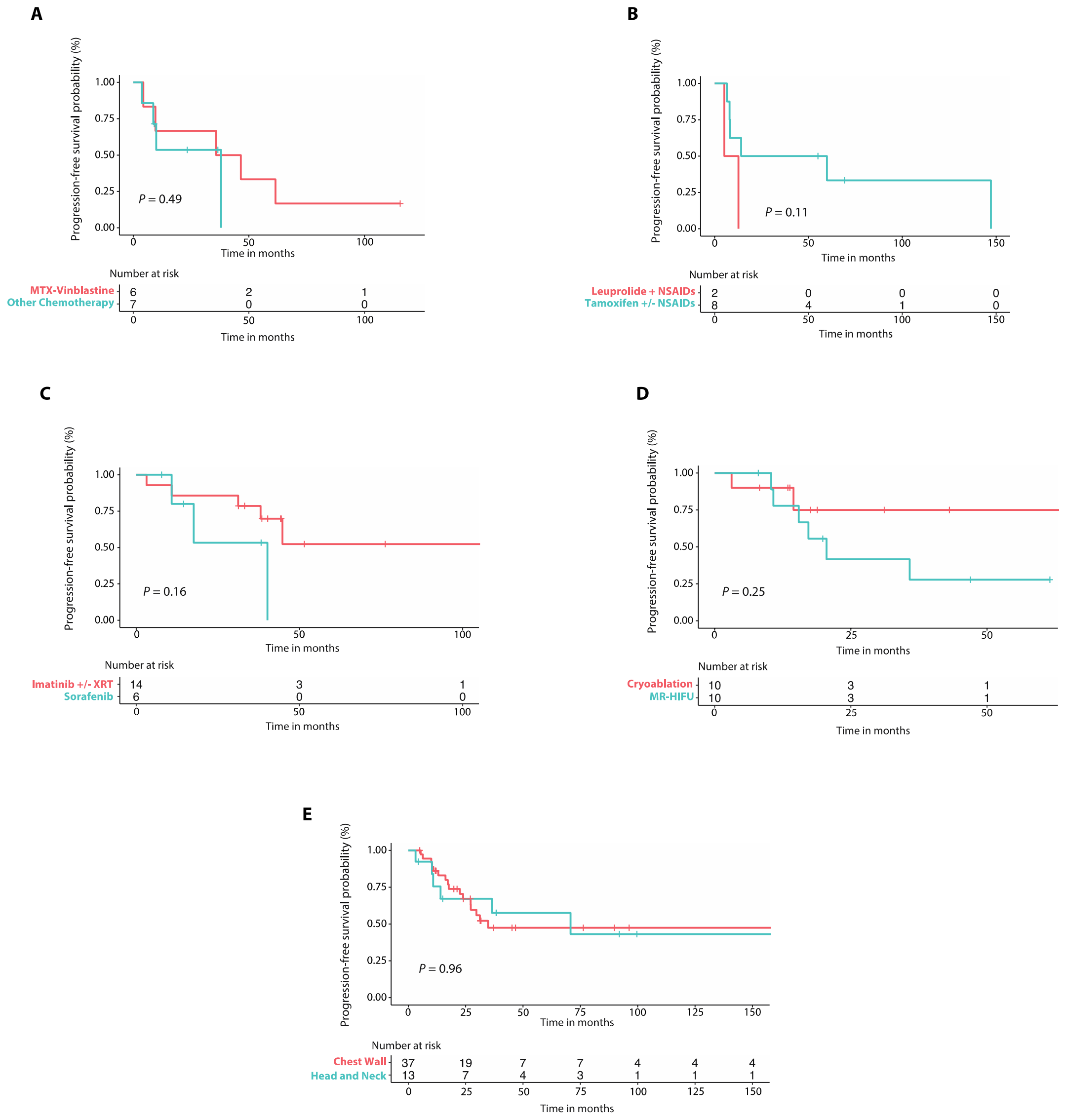

Figure S4. Stratified progression-free survival (PFS) analysis: methotrexate-vinblastine versus other chemotherapy; leuprolide with or without NSAIDs versus tamoxifen with or without NSAIDs; imatinib with or without radiotherapy versus sorafenib; Cryoablation versus MR-HIFU: chest wall versus head and neck. (A) Kaplan-Meier curves show PFS in patients with desmoid tumor treated with methotrexate-vinblastine compared to other chemotherapy regimens. (B) Kaplan-Meier curves show PFSin patients with desmoid tumor treated with leuprolide with or without NSAIDs versus tamoxifen with or without NSAIDs. (C) Kaplan-Meier curves show PFS in patients with desmoid tumor treated with imatinib compared to those treated with sorafenib. (D) Kaplan-Meier curves show PFS in patients with desmoid tumor treated with cryoablation compared to MR-HIFU. (E) Kaplan-Meier curves show PFS of patients with desmoid tumor of the chest wall compared to those of the head and neck. P-values were calculated with the Log-Rank method. MTX: Methotrexate; NSAIDs: Non-steroidal anti-inflammatory drugs; MR-HIFU: Magnetic resonance high intensity focused ultrasound; XRT: Radiotherapy.

Table S1. Progression-free survival rates after first-line treatment for curves in Figure S4.

Variables	Patients (events)	PFS	
		5-years	95% CI
MTX-Vinblastine	6(5)	33.3%	4.6–67.5%
Other Chemotherapy	7(4)	0.0%	0.0–0.0%
Sorafenib	6(3)	0.0%	0.0–0.0%
Imatinib with or without radiotherapy	14(5)	52.3%	16.1–79.5%
Leuprolide + NSAIDs	2(2)	0.0%	0.0–0.0%
Tamoxifene +/- NSAIDs	8(6)	33.3%	5.6–65.4%
Cryoablation	10(2)	77.1%	34.4–93.8%
MR-HIFU	10(6)	27.8%	4.4–59.2%
Chest Wall	37(16)	47.8%	28.7–64.6%
Head and Neck	13(6)	57.5%	25.7%–79.8%

PFS: Progression-free survival; MR-HIFU: Magnetic Resonance-guided High Intensity Focused Ultrasound; Other Chemotherapy: Doxorubicin-Dacarbazine, Doxorubicin single-agent, Docetaxel-Dacarbazine, Hydroxyurea; NSAIDs: Non-steroidal Anti-inflammatory Drugs; MTX: Methotrexate.

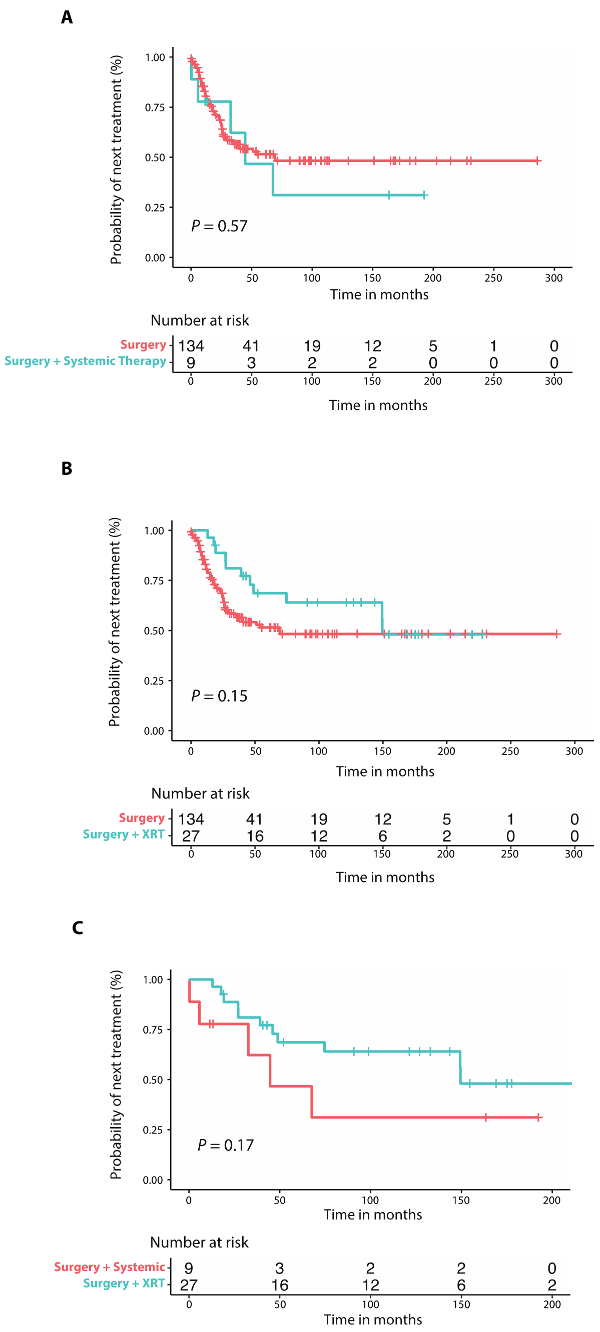

Figure S5. Time to next treatment (TTNT) among patients with desmoid tumors treated with surgery alone, surgery plus adjuvant radiotherapy or surgery plus adjuvant systemic therapy. (A) Kaplan-Meier curves show TTNT of patients treated with surgery compared to patients treated with surgery plus adjuvant systemic therapy. (B) Kaplan-Meier curves show TTNT of patients treated with surgery compared to patients treated with surgery plus adjuvant radiotherapy. (C) Kaplan-Meier curves show TTNT of patients treated with surgery plus adjuvant systemic therapy compared to surgery plus adjuvant radiotherapy. P-values were calculated with the Log-Rank method. XRT: radiotherapy.

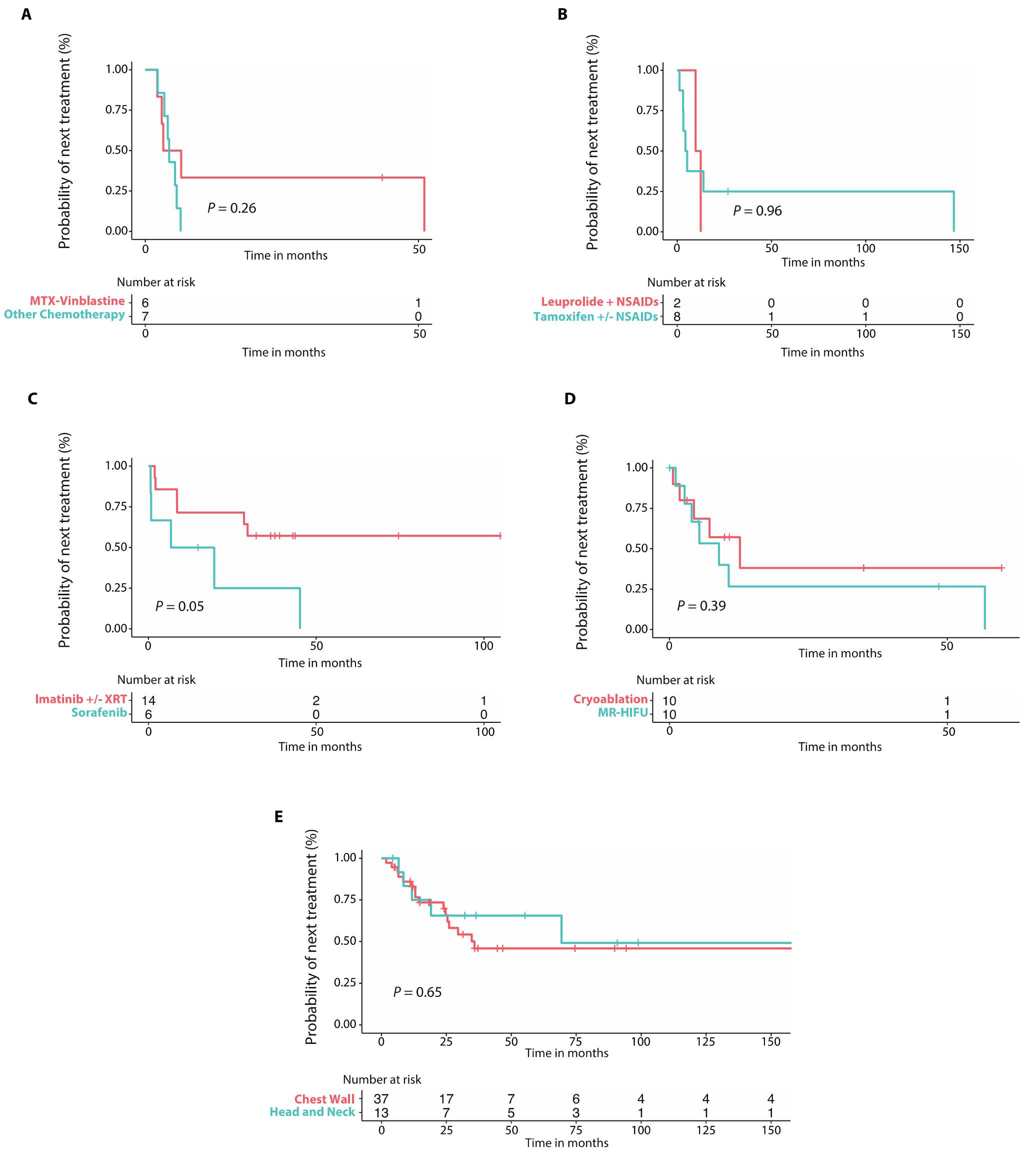

Figure S6. Stratified time to next treatment (TTNT) analysis: methotrexate-vinblastine versus other chemotherapy; leuprolide with or without NSAIDs versus tamoxifen with or without NSAIDs; imatinib with or without radiotherapy versus sorafenib; Cryoablation versus MR-HIFU; chest wall versus head and neck. (A) Kaplan-Meier curves show TTNT in patients with desmoid tumor treated with methotrexate-vinblastine compared to other chemotherapy regimens. (B) Kaplan-Meier curves show TTNT in patients with desmoid tumor treated with leuprolide with or without NSAIDs versus tamoxifen with or without NSAIDs. (C) Kaplan-Meier curves show TTNT in patients with desmoid tumor treated with imatinib compared to those treated with sorafenib. (D) Kaplan-Meier curves show TTNT in patients with desmoid tumor treated with cryoablation compared to MR-HIFU. (E) Kaplan-Meier curves show TTNT of patients with desmoid tumor of the chest wall compared to those of the head and neck. P-values were calculated with the Log-Rank method. MTX: Methotrexate; NSAIDs: Non-steroidal anti-inflammatory drugs; MR-HIFU: Magnetic resonance high intensity focused ultrasound; XRT: Radiotherapy.

Table S2. Time to next treatment after first-line treatment for curves in Figure S6.

Variables	Patients (events)	TTNT	
		Median (months)	95% CI (months)
MTX-Vinblastine	6(5)	3.3	0.0–7.6
Other Chemotherapy	7(7)	4.4	3.7–4.9
Sorafenib	6(5)	6.7	0.0–24.8
Imatinib	14(6)	Not Reached	Not Reached
Leuprolide + NSAIDs	2(2)	9.7	Not Reached
Tamoxifene +/- NSAIDs	8(7)	4.4	1.5–7.2
Cryoablation	10(5)	12.7	1.9–23.4
MR-HIFU	10(7)	8.9	2.6–15.2
Chest Wall	37(16)	35.8	Not Reached
Head and Neck	13(5)	69.3	Not Reached

TTNT: Time to next treatment; MR-HIFU: Magnetic Resonance-guided High Intensity Focused Ultrasound; Other Chemotherapy: Doxorubicin-Dacarbazine, Doxorubicin single-agent, Docetaxel-Dacarbazine, Hydroxyurea; NSAIDs: Non-steroidal Anti-inflammatory Drugs; MTX: Methotrexate.

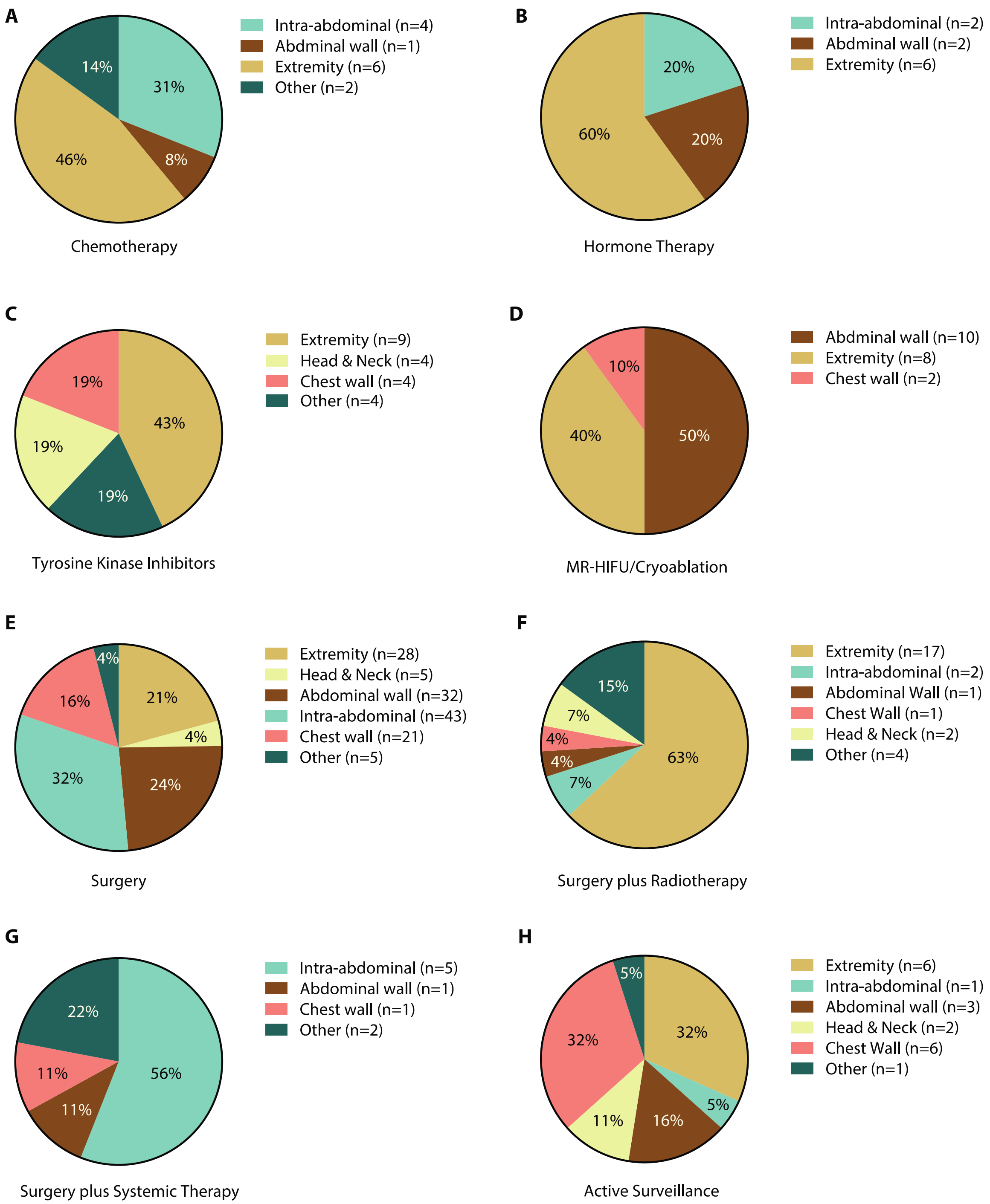

Figure S7. Distribution of tumor site in patients treated with chemotherapy (A), hormonal therapy (B), tyrosine kinase inhibitors (C), high intensity focused ultrasound (MR-HIFU) or cryoablation (D), surgery (E), surgery with adjuvant radiotherapy (F), surgery with systemic therapy (G), or active surveillance (H). Other: retroperitoneum, pelvis, scapula, para-spinal, intra-thoracic.

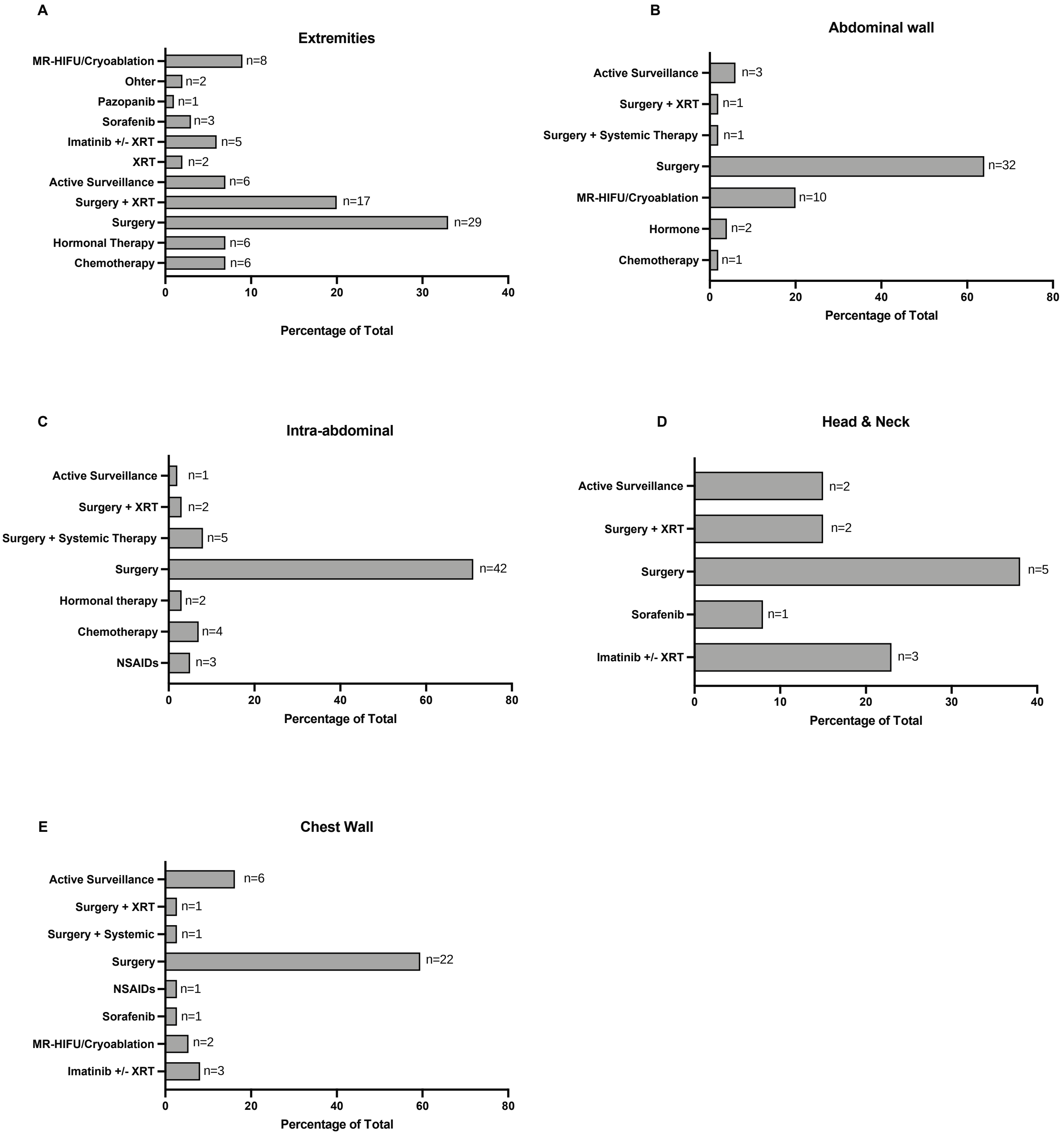

Figure S8. First-line treatment based on tumor location. Bar graphs show frequency of first-line treatments employed in desmoid tumors of the extremities (A), abdominal wall (B), those arising intra-abdominally (C), those of the head and neck (D) and those of the chest wall (E). NSAIDs: Non-steroidal anti-inflammatory drugs; XRT: radiotherapy; MR-HIFU: magnetic resonance-guided high intensity focused ultrasound.

Table S3. Progression-free survival after first-line treatment in patients with desmoid tumors of the extremities.

		**PFS**		**Univariate analysis**		**Multivariate analysis**	
**Variables**	**Patients (Events)**	1-year (95% CI)	5-year (95% CI)	HR (95% CI)	*p*-Value	HR (95% CI)	*p*-Value
**First-line Treatment**							
Surgery	29(21)	61.7% (41.6–76.6%)	22.5% (8.9–39.9%)	1.2 (0.7–2.1)	0.41	–	–
Surgery plus XRT	17(9)	100% (N/A)	63.0% (35.4–81.4%)	0.3 (0.1–0.7)	**0.006**	0.3 (0.1–0.8)	**0.01**
Chemotherapy	6(6)	33.0% (4.3–67.5%)	16.7% (0.9–51.7%)	2.0 (0.8–4.7)	0.10	–	–
Hormonal Therapy	6(6)	50.0% (11.1–80.3%)	0.0% (0.0–0.0%)	2.6 (1.1–6.1)	**0.02**	1.8 (0.7–4.8)	0.21
TKIs	9(4)	88.9% (43.3–98.4%)	30.5% (1.6–71.5%)	0.5 (0.1–1.5)	0.23	–	–
Cryoablation/MR-HIFU	8(7)	58.3% (18–84.4%)	0.0% (0.0–0.0%)	2.4 (1.0–5.5)	**0.03**	1.8 (0.7–4.2)	0.16
Active Surveillance	6(3)	62.5% (14.2–89.3%)	41.7% (5.7–7%)	0.8 (0.2–2.8)	0.84	–	–
**Age (years)**							
>40	29(16)	93.1% (75.2–98.2%)	52.1% (30.5–69.9%)	1.1 (0.6–1.9)	0.70	–	–
≤40	52(43)	56.7% (42.0–69.0%)	16.9% (7.6–29.4%)	0.9 (0.5–1.5)	0.70	1.1 (0.5–2.3)	0.73
**Sex**							
Male	22(19)	63.6% (40.3–79.8%)	21.8% (7.5–40.8%)	1.4 (0.8–2.6)	0.16	–	–
Female	59(40)	72.0% (58.4–81.8%)	32.8% (20.0–46.3%)	0.6 (0.3–1.1)	0.16	–	–
**Tumor size**							
>60 mm	49(38)	60.5% (45.3–72.7.5%)	20.7% (9.6–34.8%)	1.8 (1.0–3.2)	**0.03**	1.8 (0.8–4.0)	0.09
≤60 mm	32(21)	84.1% (66.0–93.0%)	42.5% (24.5–59.4%)	0.5 (0.3–0.9)	**0.03**	–	–
**Mutation**							
CTNNB1 p.T41A	4(4)	75.0% (13.0–96.0%)	25.0% (1.0–66.5%)	0.5 (0.1–2.1)	0.36	–	–
CTNNB1 p.S45F	5(4)	60.0% (12.6–88.2%)	40.0% (5.2–75.3%)	1.5 (0.5–4.2)	0.41	–	–

XRT: Radiotherapy; TKIs: Tyrosine-Kinase Inhibitors; MR-HIFU: Magnetic Resonance-guided high intensity focused ultrasound; Only variables that had *p*<0.05 in the univariate analysis were included in the multivariate analysis.

Table S4. Time to next treatment after first-line therapy in patients with desmoid tumors of the extremities.

		**TTNT**		**Univariate analysis**		**Multivariate analysis**	
**Variables**	**Patients (Events)**	Median (months)	(95% CI)	HR (95% CI)	*p* value	HR (95% CI)	*p* value
**First-line Treatment**							
Surgery	29(23)	17.0	5.9–28.0	0.1(0.7–2.2)	0.32	–	–
Surgery plus XRT	17(9)	149.0	54.9–243.0	0.2(0.1–0.6)	**0.001**	0.3(0.1–0.7)	**0.006**
Chemotherapy	6(6)	1.8	0.3–7.6	3.2(1.3–7.6)	**0.008**	2.7(1.1–6.5)	**0.02**
Hormonal Therapy	6(5)	10.0	0.3–19.6	1.9(0.7–5.0)	0.14	–	–
TKIs	9(6)	20.0	5.3–34.6	1.0(0.4–2.3)	0.97	–	–
Cryoablation/MR-HIFU	8(7)	9.0	0.0–19.2	2.5(1.1–5.5)	**0.02**	2.1(0.9–4.7)	0.07
Active Surveillance	6(3)	33.0	20.4–45.5	0.5(0.1–1.8)	0.36	–	–
**Age (years)**							
>40	29(14)	26.0	18.8–33.1	0.8(0.4–1.4)	0.54	–	–
≤40	52(42)	17.0	10.3–23.6	1.1(0.6–2.0)	0.54	–	–
**Sex**							
Male	22(19)	13.0	0.0–29.0	1.1(0.6–2.0)	0.56	–	–
Female	59(37)	20.0	10.5–29.4	0.8(0.4–1.4)	0.56	–	–
**Tumor size**							
>60 mm	49(38)	17.0	6.7–27.3	1.3(0.8–2.3)	0.23	–	–
≤60 mm	32(18)	21.0	11.7–30.2	0.7(0.4–1.2)	0.23	–	–
**Mutation**							
CTNNB1 p.T41A	4(4)	25.0	0.0–53.4	0.9(0.3–2.7)	0.96	–	–
CTNNB1 p.S45F	5(4)	9.0	0.4–17.5	1.4(0.5–4.1)	0.44	–	–

XRT: Radiotherapy; TKIs: Tyrosine-Kinase Inhibitors; MR-HIFU: Magnetic Resonance-guided high intensity focused ultrasound; Only variables that had $p<0.05$ in the univariate analysis were included in the multivariate analysis.

Table S5. Progression-free survival after first-line treatment in patients with desmoid tumors of the abdominal wall.

		PFS		Univariate analysis	
**Variables**	**Patients (Events)**	1-year (95% CI)	5-year (95% CI)	HR (95% CI)	*p*-Value
**First-line Treatment**					
Surgery	32(12)	90.5% (73.4–96.8%)	63.5% (43.8–77.9%)	1.9 (0.4–8.7)	0.38
Cryoablation/MR-HIFU	10(0)	100% (N/A)	100% (N1/A)	0.03 (0.0–11.8)	0.26
Hormonal Therapy	2(1)	50% (0.6–91.0%)	50% (0.6–91.0%)	1.9 (0.2–15.3)	0.50
Active surveillance	3(1)	100% (N/A)	0.0% (0.0–0.0%)	4.2 (0.4–36.7)	0.19
**Age (years)**					
>40	16(6)	100% (N/A)	71.4% (40.7–88.1%)	1.3 (0.4–3.9)	0.55
≤40	31(8)	86.9% (68.9–94.8%)	64.9% (42.7–80.3%)	0.7 (0.2–2.0)	0.55
**Sex**					
Male	7(4)	100% (N/A)	71.4% (25.8–91.9%)	2.3 (0.7–7.4)	0.15
Female	40(10)	89.7% (74.9–96.0%)	66.8% (47.7–80.2%)	0.4 (0.1–1.3)	0.15
**Tumor size**					
>60 mm	16(5)	93.7% (63.2–99.0%)	60.5% (29.0–81.5%)	1.1 (0.3–3.4)	0.82
≤60 mm	31(9)	89.9% (72.0–96.5%)	70.6% (49.5–84.1%)	0.8 (0.2–2.6)	0.82
**Mutation**					
CTNNB1 p.T41A	6(2)	83.3% (27.3–97.4%)	55.5% (7.3–87.5%)	1.9 (0.4–9.0)	0.37

MR-HIFU: Magnetic Resonance-guided high intensity focused ultrasound; A multivariate analysis was not performed given that there were no variables with $p<0.05$ in the univariate analysis.

Table S6. Time to next treatment after first-line therapy in patients with desmoid tumors of the abdominal wall.

		TTNT		Univariate analysis		Multivariate analysis	
**Variables**	**Patients (Events)**	Median (months)	(95% CI)	HR (95% CI)	*p* value	HR (95% CI)	*p* value
**First-line Treatment**							
Surgery	32(10)	Not reached	Not reached	0.3(0.1–0.9)	**0.03**	0.8(0.2–3.0)	0.74
Cryoablation/MR-HIFU	10(4)	Not reached	Not reached	2.1(0.6–6.7)	0.19	–	–
Hormonal Therapy	2(2)	3.0	Not reached	13.1(2.3–72.9)	**0.003**	14.9(1.8–120.6)	**0.01**
Active surveillance	3(1)	170.0	Not reached	0.9(0.1–7.3)	0.95	–	–
**Age (years)**							
>40	16(4)	170.0	0.0–370.0	0.4(0.1–1.4)	0.17	–	–
≤40	31(13)	69.0	Not reached	2.1(0.7–6.7)	0.17	–	–
**Sex**							
Male	7(3)	Not reached	Not reached	1.2(0.3–4.4)	0.71	–	–
Female	40(14)	170.0	Not reached	0.7(0.2–2.7)	0.71	–	–
**Tumor size**							
>60 mm	16(5)	Not reached	Not reached	0.9(0.3–2.6)	0.88	–	–
≤60 mm	31(12)	170.0	0.0–373.8	1.0(0.3–3.0)	0.88	–	–
**Mutation**							
CTNNB1 p.T41A	6(4)	6.0	0.0–106.2	3.4(1.0–10.8)	**0.03**	3.7(0.8–15.9)	0.07

MR-HIFU: Magnetic Resonance-guided high intensity focused ultrasound; Only variables that had $p<0.05$ in the univariate analysis were included in the multivariate analysis.

Table S7. Progression-free survival after first-line treatment in patients with intra-abdominal desmoid tumors.

		PFS		Univariate analysis	
**Variables**	**Patients (Events)**	1-year (95% CI)	5-year (95% CI)	HR (95% CI)	*p*-Value
**First-line Treatment**					
Surgery	42(11)	92.3% (78.0–97.4%)	68.2% (49.5–81.1%)	0.5 (0.1–1.4)	0.20
Surgery plus XRT	2(1)	100% (N/A)	50.0% (0.6–91.0%)	1.2 (0.1–9.3)	0.83
Surgery plus Systemic Therapy	5(3)	100% (N/A)	75.0% (12.8–96.0%)	1.9 (0.5–7.0)	0.28
Hormonal Therapy	2(1)	100% (N/A)	100% (N/A)	0.04 (0.0–16505.6)	0.63
Chemotherapy	4(2)	75.0% (12.8–96.0%)	75.0% (12.8–96.0%)	2.6 (0.5–12.0)	0.20
**Age (years)**					
>40	29(14)	92.0% (71.5–97.9%)	72.7% (86.8–48.7%)	0.8 (0.3–2.3)	0.82
≤40	26(6)	92.3% (72.6–98.0%)	65.1% (41.4–81.1%)	1.1 (0.4–2.9)	0.82
**Sex**					
Male	29(10)	100% (N/A)	69.9% (46.6–84.5%)	1.9 (0.6–5.6)	0.19
Female	26(7)	83.8% (62.4–93.5%)	67.7% (43.1–83.4%)	0.5 (0.1–1.4)	0.19
**Tumor size**					
>60 mm	33(12)	93.9% (77.9–98.4%)	20.7% (9.6–34.8%)	2.0 (0.6–6.3)	0.20
≤60 mm	22(5)	63.9% (42.6–79.0%)	77.9% (51.1–91.1%)	0.4 (0.1–1.4)	0.20
**Mutation**					
CTNNB1 p.T41A	5(0)	100% (N/A)	100% (N/A)	0.04 (0.0–84.8)	0.41

XRT: Radiotherapy; A multivariate analysis was not performed given that there were no variables with $p<0.05$ in the univariate analysis.

Table S8. Time to next treatment after first-line therapy in patients with intra-abdominal desmoid tumors.

		TTNT		Univariate analysis	
**Variables**	**Patients (Events)**	Median (months)	(95% CI)	HR (95% CI)	*p* value
**First-line Treatment**					
Surgery	42(13)	Not reached	Not reached	0.4(0.1–1.2)	0.12
Surgery plus XRT	2(0)	N/A	N/A	0.04(0.0–379.9)	0.50
Surgery plus Systemic Therapy	5(3)	68.0	0.0–164.7	1.9(0.5–6.8)	0.27
Hormonal Therapy	2(1)	6.0	Not reached	1.8(0.1–14.3)	0.54
Chemotherapy	4(3)	3.0	0.0–25.0	3.6(1.0–12.7)	**0.04**
**Age (years)**					
>40	29(10)	Not reached	Not reached	0.8(0.3–1.9)	0.64
≤40	26(10)	147.0	32.2–261.7	1.2(0.5–2.9)	0.64
**Sex**					
Male	29(12)	Not reached	Not reached	1.1(0.4–2.9)	0.70
Female	26(8)	147.0	0.0–314.3	0.8(0.3–2.0)	0.70
**Tumor size**					
>60 mm	33(14)	Not reached	Not reached	1.3(0.5–3.6)	0.49
≤60 mm	22(6)	147.0	0.0–314.3	0.7(0.2–1.8)	0.49
**Mutation**					
CTNNB1 p.T41A	5(3)	6.0	0.0–12.4	3.0(0.8–10.7)	0.08

XRT: Radiotherapy; A multivariate analysis was not performed given that there was only one variable with $p<0.05$ in the univariate analysis.

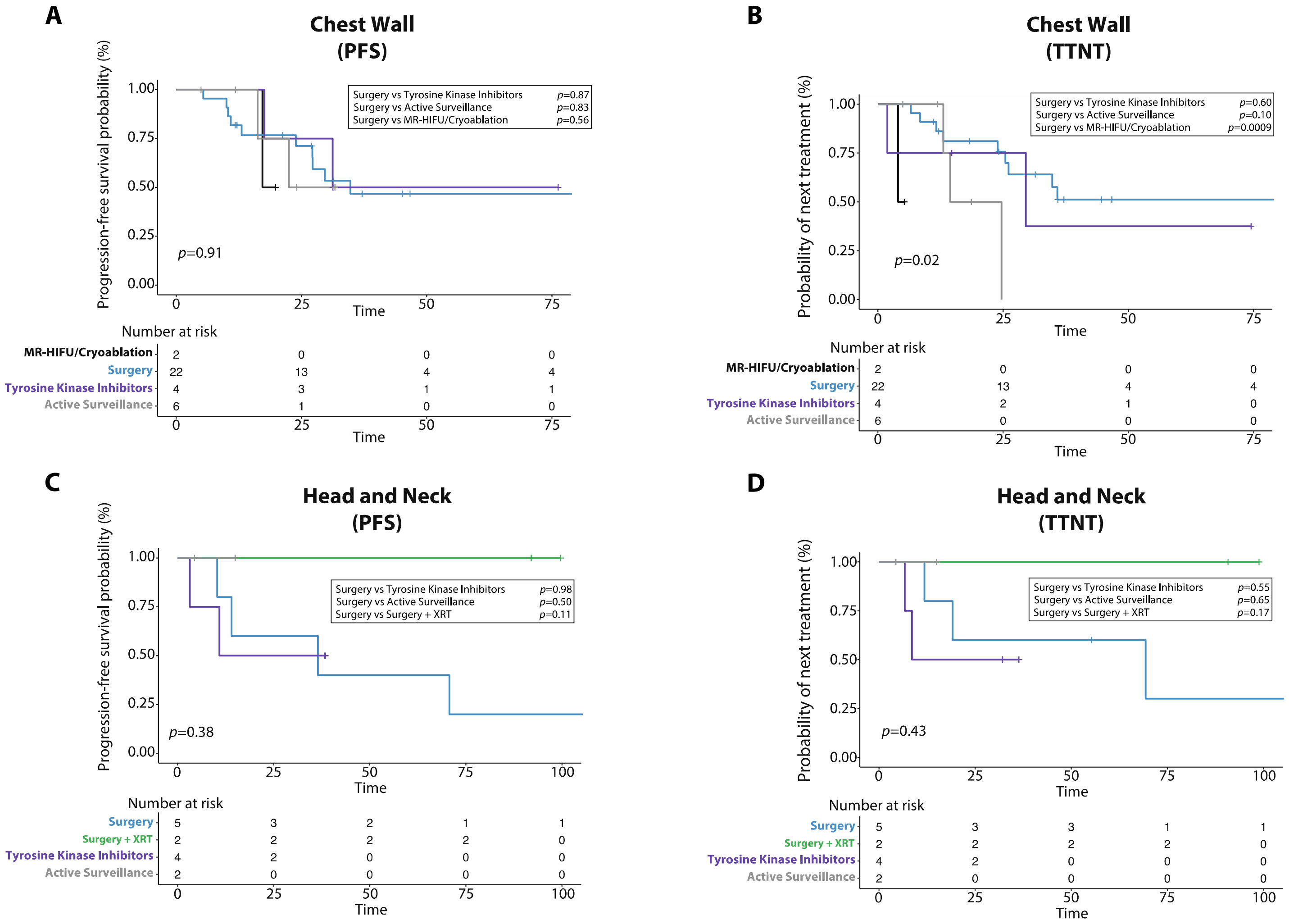

Figure S9. Progression-free survival (PFS) and time to next treatment (TTNT) after first line treatment in desmoid tumors (DTs) of the chest wall and head and neck. (A) Kaplan-Meier curve shows PFS after first-line treatment in DTs of the chest wall. (B) Kaplan-Meier curve shows TTNT after first-line treatment in DTs of the chest wall. (C) Kaplan-Meier curve shows PFS after first-line treatment in DTs of the head and neck. (D) Kaplan-Meier curve shows TTNT after first-line treatment in DTs of the head and neck. XRT: radiotherapy; MR-HIFU: magnetic resonance-guided high intensity focused ultrasound.

Table S9. Progression-free survival after first-line therapy in patients with desmoid tumors of the chest wall.

		PFS		Univariate analysis	
**Variables**	**Patients (Events)**	1-year (95% CI)	5-year (95% CI)	HR (95% CI)	*p*-*V*alue
**First-line Treatment**					
Surgery	22(10)	81.8% (58.6–92.7%)	47.4% (24.0–68.2%)	0.8 (0.3–2.6)	0.84
Cryoablation/MR-HIFU	2(1)	100% (N/A)	50.0% (0.6–91.0%)	2.0 (0.2–16.3)	0.51
Tyrosine Kinase Inhibitors	4(2)	100% (N/A)	50.0% (5.2–84.4%)	0.8 (0.1–3.6)	0.79
Active surveillance	6(2)	100% (N/A)	50.0% (5.2–84.4%)	1.1 (0.2–5.3)	0.83
**Age (years)**					
>40	16(10)	87.0% (96.5–57.3%)	71.8% (40.8–88.4%)	2.2 (0.7–6.5)	0.14
≤40	18(5)	88.8% (62.4–97.0%)	14.9% (1.1–44.6%)	0.4 (0.1–1.3)	0.14
**Sex**					
Male	6(4)	83.3% (27.3–97.4%)	55.5% (7.3–87.5%)	2.6 (0.8–8.5)	0.09
Female	28(11)	89.2% (70.3–96.3%)	44.0% (22.7–63.4%)	0.3 (0.1–1.1)	0.09
**Tumor size**					
>60 mm	12(6)	83.3% (48.2–95.5%)	48.6% (19.2–72.9%)	1.3 (0.4–3.8)	0.54
≤60 mm	22(9)	90.4% (67.0–97.4%)	44.4% (19.1–67.1%)	0.7 (0.2–2.0)	0.54
**Mutation**					
CTNNB1 p.T41A	3(2)	96.8% (79.8–99.4%)	92.6% (73.3–98.0%)	1.5 (0.3–6.8)	0.57
APC	2(0)	100% (N/A)	92.8% (59.1–98.9%)	0.04 (0.0–590.2)	0.52

MR-HIFU: Magnetic Resonance-guided high intensity focused ultrasound; A multivariate analysis was not performed given that there were no variables with $p<0.05$ in the univariate analysis.

Table S10. Time to next treatment after first-line therapy in patients with desmoid tumors of the chest wall.

		TTNT		Univariate analysis		Multivariate analysis	
**Variables**	**Patients (Events)**	Median (months)	(95% CI)	HR (95% CI)	*p* value	HR (95% CI)	*p* value
**First-line Treatment**							
Surgery	22(9)	Not reached	Not reached	0.4(0.1–1.2)	0.11	–	–
Cryoablation/MR-HIFU	2(1)	4.0	Not reached	15.7(0.9–251.7)	0.05	–	–
Tyrosine Kinase Inhibitors	4(2)	30.0	0.0–71.9	1.1(0.2–5.1)	0.84	–	–
Active surveillance	6(3)	14.0	6.1–21.8	2.3(0.6–9.2)	0.21	–	–
**Age (years)**							
>40	16(11)	25.0	23.2–26.7	3.5(1.1–11.3)	**0.03**	2.5(0.5–11.5)	0.22
≤40	18(4)	Not reached	Not reached	0.2(0.0–0.8)	**0.03**	–	–
**Sex**							
Male	6(4)	13.0	0.0–29.3	2.3(0.7–7.4)	0.15	–	–
Female	28(11)	36.0	Not reached	0.4(0.1–1.3)	0.15	–	–
**Tumor size**							
>60 mm	12(8)	13.0	0.0–29.9	3.0(1.0–8.4)	**0.03**	1.6(0.4–6.1)	0.48
≤60 mm	22(7)	Not reached	Not reached	0.3(0.1–0.9)	**0.03**	–	–
**Mutation**							
CTNNB1 p.T41A	3(1)	Not reached	Not reached	0.4(0.0–3.2)	0.40	–	–
APC	2(0)	N/A	N/A	0.04(0.0–466.4)	0.51	–	–

MR-HIFU: Magnetic Resonance-guided high intensity focused ultrasound; Only variables that had $p<0.05$ in the univariate analysis were included in the multivariate analysis.

Table S11. Progression-free survival after first-line therapy in patients with desmoid tumors of the head and neck.

		PFS		Univariate analysis	
**Variables**	**Patients (Events)**	1-year (95% CI)	5-year (95% CI)	HR (95% CI)	*p*-Value
**First-line Treatment**					
Surgery	5(4)	80.0% (20.4–96.9%)	40.0% (5.2–75.2%)	2.6 (0.4–14.3)	0.27
Surgery plus XRT	2(0)	100% (N/A)	100% (N/A)	0.02 (0.0–75.0)	0.37
Tyrosine Kinase Inhibitors	4(2)	50% (5.8–84.4%)	50% (5.8–84.4%)	1.7 (0.2–10.4)	0.54
Active surveillance	2(0)	100% (N/A)	100% (N/A)	0.04 (0.0–30734.5)	0.64
**Age (years)**					
>40	8(3)	72.9% (27.7–92.5%)	72.9% (27.7–92.5%)	0.8 (0.1–4.1)	0.81
≤40	5(3)	80.0% (20.4–96.9%)	40.0% (5.2–75.2%)	1.2 (0.2–6.0)	0.81
**Sex**					
Male	2(2)	50.0% (0.6–91.0%)	50.0% (0.6–91.0%)	3.1 (0.5–17.7)	0.19
Female	11(4)	80.0% (40.9–94.5%)	58.3% (22.8–82.0%)	0.3 (0.0–1.7)	0.19
**Tumor size**					
>60 mm	8(3)	87.5% (39.5–98.1%)	75.0% (31.5–93.0%)	0.2 (0.0–1.7)	0.18
≤60 mm	5(3)	50.0% (5.8–84.4%)	25.0% (0.9–66.5%)	3.3 (0.5–20.6)	0.54
**Mutation**					
CTNNB1 p.T41A	2(2)	50.0% (0.6–91.0%)	0.0% (0.0–0.0%)	3.6 (0.5–21.8)	0.16

XRT: Radiotherapy; A multivariate analysis was not performed given that there were no variables with p<0.05 in the univariate analysis.

Table S12. Time to next treatment after first-line therapy in patients with desmoid tumors of the head and neck.

		TTNT		Univariate analysis	
**Variables**	**Patients (Events)**	Median (months)	(95% CI)	HR (95% CI)	*p* value
**First-line Treatment**					
Surgery	5(3)	69.0		1.6(0.2–10.0)	0.57
Surgery plus XRT	2(0)	N/A	N/A	0.02(0.0–157.1)	0.41
Tyrosine Kinase Inhibitors	4(2)	9.0	Not reached	2.8(0.3–20.3)	0.30
Active surveillance	2(0)	N/A	N/A	0.0(0.0–1369077.0)	0.72
**Age (years)**					
>40	8(3)	69.0	0.0–152.3	1.2(0.2–7.5)	0.80
≤40	5(2)	Not reached	Not reached	0.8(0.1–4.8)	0.80
**Sex**					
Male	2(1)	69.0	0.0–29.3	0.9(0.1–8.4)	0.95
Female	11(4)	Not reached	Not reached	1.0(0.1–9.5)	0.95
**Tumor size**					
>60 mm	8(2)	Not reached	Not reached	0.09(0.0–0.9)	**0.04**
≤60 mm	5(3)	9.0	4.1–13.9	11.0(1.0–111.0)	**0.04**
**Mutation**					
CTNNB1 p.T41A	2(1)	9.0	Not reached	2.0(0.2–19.5)	0.54

XRT: Radiotherapy; A multivariate analysis was not performed given that there was only one variable with $p<0.05$ in the univariate analysis.
